# ChIAMM: A Mixture Model for Statistical Analysis of Long-Range Chromatin Interactions From ChIA-PET Experiments

**DOI:** 10.3389/fgene.2020.616160

**Published:** 2020-12-14

**Authors:** Yibeltal Arega, Hao Jiang, Shuangqi Wang, Jingwen Zhang, Xiaohui Niu, Guoliang Li

**Affiliations:** ^1^Agricultural Bioinformatics Key Laboratory of Hubei Province, Hubei Engineering Technology Research Center of Agricultural Big Data, College of Informatics, Huazhong Agricultural University, Wuhan, China; ^2^National Key Laboratory of Crop Genetic Improvement, Huazhong Agricultural University, Wuhan, China

**Keywords:** ChIA-PET, chromatin interactions, genome-wide, mixture model, bayesian framework

## Abstract

Chromatin interaction analysis by paired-end tag sequencing (ChIA-PET) is an important experimental method for detecting specific protein-mediated chromatin loops genome-wide at high resolution. Here, we proposed a new statistical approach with a mixture model, chromatin interaction analysis using mixture model (ChIAMM), to detect significant chromatin interactions from ChIA-PET data. The statistical model is cast into a Bayesian framework to consider more systematic biases: the genomic distance, local enrichment, mappability, and GC content. Using different ChIA-PET datasets, we evaluated the performance of ChIAMM and compared it with the existing methods, including ChIA-PET Tool, ChiaSig, Mango, ChIA-PET2, and ChIAPoP. The result showed that the new approach performed better than most top existing methods in detecting significant chromatin interactions in ChIA-PET experiments.

## Introduction

Diverse high-throughput methods have been developed to detect genome-wide chromatin interactions, including chromatin interaction analysis by paired-end tag sequencing (ChIA-PET) and high-throughput chromosome conformation capture (Hi-C) (Fullwood et al., 2009; Lieberman-Aiden et al., 2009). ChIA-PET was first introduced in 2009 as an essential experimental method for studying genome-wide chromatin interactions mediated by a specific protein of interest. It can discover many chromatin interactions at a higher resolution that are needed for studying gene transcription regulation. It has been widely used to study various proteins such as estrogen receptor alpha, RNA polymerase II (RNAPII), CCCTC binding factor (CTCF) in human and mouse genome (Fullwood et al., 2009; Handoko et al., 2011; Li et al., 2012; Tang et al., 2015), and H3K4me3, H3K9me2, and RNAPII in rice and maize (Peng et al., 2019; Zhao et al., 2019).

The processing of raw ChIA-PET data is not easy. ChIA-PET experiment will generate tens of millions of paired reads containing a tag and linker sequence (barcode). The tag can be short (generated by the original protocol, and it is about 20 base pairs) or long (generated by the improved protocol, and it is about 150–250 base pairs) (Li et al., 2017). The steps to process raw ChIA-PET data include linker trimming, read alignment, paired-end tag (PET) filtering, PCR duplicate removal, peak calling, and chromatin interaction calling. In ChIA-PET data, similar to other high-throughput sequencing data, there is a mixture of signals (fragment pairs from real chromatin interactions, termed as true pairs) and noise (fragment pairs from random ligation, termed as false pairs). Distinguishing the true interaction pairs from the random noise is not a simple task, and complicated computational tools are needed (He et al., 2016). Up to now, there are several published tools, and ChIA-PET Tool (Li et al., 2010), ChiaSig (Paulsen et al., 2014), Mango (Phanstiel et al., 2015), ChIA-PET2 (Li et al., 2016), and ChIAPoP (Huang et al., 2019) are the representative ones.

The ChIA-PET Tool is the first software package for the automatic processing of ChIA-PET sequence data, which uses hypergeometric distribution (HG) as the statistical method and accounts for the sequencing depth bias. It fails to correct the major source of bias (He et al., 2015; Phanstiel et al., 2015), such as the genomic distance between the interacting regions. ChiaSig (Paulsen et al., 2014) advanced the ChIA-PET Tool by incorporating genomic distance between interacting anchors. It uses non-central HG distribution for modeling the frequency of chromatin interactions, and the model considers the non-specific ligations that exist because of genomic distance proximity. As a limitation, ChiaSig has a high false-negative rate (He et al., 2015), it executes the final step in ChIA-PET Tool data analysis, and users are expected to write their programs (Phanstiel et al., 2015). Similar to ChiaSig, Mango (Phanstiel et al., 2015) is designed for correcting the primary source of biases from genomic proximity using the binomial model. As a limitation, Mango does not model the interactions between different chromosomes. Besides, it is too conservative at the significant loop calling step, just reporting a small number of interactions, which led to a high false-negative rate (Li et al., 2016). ChIA-PET2 (Li et al., 2016) is a complete analysis pipeline that uses a Bayesian mixture model to process both bridge and half-linker ChIA-PET data from raw sequencing reads to significant chromatin loop calls. As a limitation, it gives slightly different results for the same input (Huang et al., 2019). ChIAPoP (Huang et al., 2019) was proposed using zero truncated Poisson distribution for accounting for the genomic distance and sequence biases. It is designed for short-read ChIA-PET datasets only. ChIAPoP considers intra- and interchromosomal interaction as a separate model. Recently, ChIA-PIPE (Lee et al., 2020) was proposed by integrating the special functions related to the experiment types, data processing, and structural interpretation. ChIA-PIPE used ChiaSig (Paulsen et al., 2014) to calculate the statistical significance of interactions.

All the above existing tools considered only the genomic distance or anchor depth as biases. But in different studies, the GC content and mappability score are listed as systematic sources of biases (Yaffe and Tanay, 2011; Hu et al., 2012; Imakaev et al., 2012). Hence, the existing tools failed to address it. Besides, from the existing tools, except for ChIA-PET Tool V3 (Li et al., 2019), ChIA-PET2 (Li et al., 2016), and ChIA-PIPE (Lee et al., 2020), others are designed exclusively for short-read ChIA-PET data analysis.

Here, we present a new statistical method called chromatin interaction analysis using mixture model (ChIAMM) to distinguish signals from noise in ChIA-PET data. It considers the genomic distance between anchors, sequence depth, GC content, and mappability as systematic sources of bias. The model was tested on both RNAPII and CTCF ChIA-PET data from human K562 and MCF7 and RNAPII and H3K9me2 ChIA-PET data from rice MH63. The performance of the proposed method was evaluated with the aggregate peak analysis (APA) plot, CTCF coverage of anchors, and CTCF motif orientation analysis. The results showed that the new method performed better with the most top existing tools.

## Materials and Methods

### Public Datasets Used

In this study, MCF7 and K562 RNAPII data in Li et al. (2012), MCF7 and K562 CTCF data in GEO with accession numbers GSM970215 and GSM970216, respectively, and MH63 RNAPII and H3K9me2 data in Zhao et al. (2019) were processed. For the CTCF enrichment and motif orientation analyses, the CTCF peak regions from ENCODE ChIP-Seq datasets ENCFF990LUT and ENCFF720OXG for MCF7, and ENCFF559HEE and ENCFF681OMH for K562 datasets were used.

### Systematic Biases Considered in the Study

In this study, we used genomic distance, GC content, mappability, and enrichment as systematic biases of the ChIA-PET experiment. We used ChIA-PET Tool version 3 (V3) (Li et al., 2019) as the primary processing pipeline to find the anchor sites, genomic distance, interaction frequency, type of interaction, marginal count, and self-ligation PETs. It is known that regions close together along the genomic sequence will have a higher chance of forming random contacts. Thus, it is essential to integrate the genomic distance into the model (Paulsen et al., 2014), and we primarily considered the genomic distance as a bias. The second bias is the GC content, defined as the percentage of cytosine (C) and guanine (G) bases in a given region. In different studies, GC content has been reported as a systematic bias in next-generation sequencing (NGS) applications (Yaffe and Tanay, 2011; Hu et al., 2012), and the GC content of each anchor is calculated using bedtools nuc (Quinlan and Hall, 2010) function. The third bias is the mappability score, which is defined as the mappability of all possible k-mers in a given anchor site. The mappability track is downloaded from the UCSC Genome Browser website (Derrien et al., 2012), and the overlap of the mappability track with anchors was performed using bedtools. The last systematic bias is the local enrichment in a given region. It is well known that the anchors with more enrichment have a higher probability of forming interligation PETs by random chance. Different studies have considered enrichment as systematic bias in their analysis (Li et al., 2010, 2016; Paulsen et al., 2014; He et al., 2015; Niu and Lin, 2015; Phanstiel et al., 2015; Huang et al., 2019). In this study, we measured the anchor enrichment using the number of self-ligation PETs found by ChIA-PET Tool (V3).

### Statistical Mixture Model

In many situations, like the ChIA-PET experiment, due to the complex nature of the observed data, using single parametric distribution is insufficient for inference. Here, we used a mixture model. It offers a solution to this problem by assuming that the frequency of chromatin interactions can be represented by a weighted sum of distributions, with each distribution representing a proportion contribution to the data.

We used a mixture model for modeling the interaction frequency of the ChIA-PET experiment. Let *Y* = {*y*_*i*_,*i* = 1,2,…….,*n*} represent the interaction frequencies for each observed anchor pair *i* from *n* unique anchor pairs (say, anchor *A*_*i*_*andB*_*i*_). The interaction frequency, *y*_*i*_, has a two-component mixture distribution, i.e., signal and noise. The mixture model integrates signal and noise interaction frequency as follows:

$$y_{i}∼\sum_{j=0}^{1}W_{j\,i}p\left(.|\lambda_{j\,i}\right)\;i=1,2,\ldots \ldots ..,n$$

where *W*_*ji*_ is the mixing probability (i.e., *W*_*0i*_ and *W*_*1i*_ represent the probability of pair *i* being a false pair and true pair, respectively), and *W*_0*i*_ + *W*_1*i*_ = 1.

It is well known that Poisson distribution is the most popular distribution for modeling NGS count data, and in the above model, *p*(.|λ) is the (*k*−1) truncated Poisson distribution. The model considers the interaction frequency, *y*_*i*_≥*k* (where *k* is a cut-off point). The cut-off point is used to decide a pair that is kept in the analysis. Most of the time, it is determined by the researcher. In this study, the cut-off value is ≥2, the same as in (Fullwood et al., 2009).

The probability mass function for Poisson distribution is written as:

$$p\left(Y=y|\lambda \right)=\frac{e^{-\lambda }\,\lambda^{y}}{y!}$$

and the probability mass function for *k-1* truncated Poisson distribution is written as follow:

$$p\left(Y=y|y\geq k,\lambda \right)=\frac{\lambda^{y}}{y!\,\left\{e^{\lambda }-\left[1+\lambda +\frac{\lambda^{2}}{2!}+\cdots +\frac{\lambda^{k-1}}{\left(k-1\right)!}\right]\right\}}$$

for⁢y=k,k+1,….

Therefore, for *k*≥2, *p*(*Y* = *y*|λ) is written as:

$$p\left(Y=y|y>1,\lambda \right)=\frac{\lambda^{y}}{y!\,\left[e^{\lambda }-\left(1+\lambda \right)\right]}\;\text{for}y=2,3,\ldots \ldots .$$

In simplified form, we can express it using the cumulative distribution function (CDF) as follows:

$$p\left(Y=y|y>1,\lambda \right)=\frac{p\,\left(y|\lambda \right)}{1-F\,\left(1\right)}$$

For pair of *i*, 1≤*i*≤*n*,*p*(*Y* = *y*|λ) will be *p*(.|λ_0*i*_) and *p*(.|λ_1*i*_), which model the interaction frequency conditional on it being noise and signal, respectively, and *F*(1) = *F*(*y*≤1) represents the probability that the random variable takes a value ≤1. Besides, from the biological perspectives, the signals have more intensity than the noises (Rousseau et al., 2011), and thus, we put the requirements λ_0*i*_ < λ_1*i*_.

From the listed biases, genomic distance has no explicit rule to measure in interchromosomal interaction data. Hence, we model the intra- and interchromosomal interaction data separately and have different rate parameters (λ) and biases (*x*_*i*_) as well. The rate parameters of intra- (λ)and inter chromosomal (λ′) interactions are connected with the biases using the link function. The listed biases in this study are GC percentage(xig⁢candx)ig′⁢c, mappability (xim⁢a⁢pandx)im′⁢a⁢p and enrichment (xie⁢n⁢randx)ie′⁢n⁢r for intra- and interchromosomal interaction, respectively. We considered the genomic distance only for intrachromosomal interactions. In the intrachromosomal analysis, we considered all the biases, but in the interchromosomal interaction analysis, we will remove out the distance from the statistical model.

The link functions of intra- and interchromosomal interaction are written as follows, respectively:

$$\begin{array}{cc} l\,o\,g\,\left(\lambda_{0\,i}\right)= & \beta_{0}+\beta_{1}\,l\,o\,g\,\left(x_{i}^{e\,n\,r}\right)+\beta_{2}\,l\,o\,g\,\left(x_{i}^{g\,c}\right)+\beta_{3}\,l\,o\,g\,\left(x_{i}^{m\,a\,p}\right)\\ & +\beta_{4}\,l\,o\,g\,\left(x_{i}^{d\,i\,s}\right) \end{array}$$

log(λ0⁢i′)=β0′+β1′log(x′)ie⁢n⁢r+β2′log(x′)ig⁢c+β3′log(x′)im⁢a⁢p

In Bayesian inference, the prior distribution is a crucial part, representing the information about an uncertain parameter. The priors and model description of inter- and intrachromosomal interactions are similar. We used the prime symbol (′) for parameters in the interchromosomal interaction model. To simplify the next discussion, we will use the intrachromosomal interaction model parameters as an example.

A normal distribution is a natural prior choice for *β*_*j*_. Therefore, the coefficients of the Poisson regression model, *β*_*j*_,*j* = 1,2,3,4 have normal prior with mean zero and reasonable variance to enable large enough deviations,*β*_*j*_∼*N*(0, 3^2^) (Carlin and Louis, 2008; Gelman et al., 2013; Halla-aho, 2015), and we declared *λ*_1*i*_ = *C* + *λ*_0*i*_ to show that the frequency of signal is greater than the noise, where *C* is a positive number that follows zero truncated normal distribution with reasonable variance, *C*∼*N*(0, 3^2^). In (Halla-aho, 2015), different *C*_*i*_ were considered, but the estimated *C*_*i*_ has very small variance. Therefore, the researcher recommended others to use the same *C* for next work. This help us in the side of reducing computational time. The statistical approach considers the correlation between common anchor pairs (Niu and Lin, 2015). The dependency incorporated in the weights of the mixture model, i.e., the weight changes from common to pair-specific values, *W*_1*i*_∼*Beta*(*mc*_*i*_,*mc*), where *mc*_*i*_ and *mc* is the marginal count of the *i*-th paired anchors and the mean of marginal count, respectively.

**FIGURE 1:**
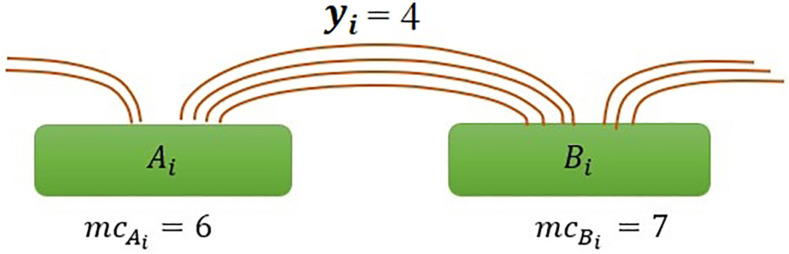
Illustration of interaction frequency in the ChIAMM model. A_i_ and B_i_ represent anchor regions with marginal PET counts *mc*_*A_i*_ and *mc*_*B_i*_ respectively, and *y*_*i*_ is the number of inter-ligation PETs between specified anchors *A*_*i*_ and *B*_*i*_.

When we compute the marginal count, we considered the interaction frequency *y*_*i*_ two times; hence, we subtracted one *y*_*i*_, i.e.,

$$m\,c_{i}=m\,c_{A_{i}}+m\,c_{B_{i}}-y_{i}$$

where *mc*_*Ai*_*andmc*_*Bi*_ are the marginal count of anchor *A*_*i*_ and *B*_*i*_, respectively, and *y*_*i*_ is the interaction count between anchors (Figure 1), and *mc* is the average of marginal counts and calculate as $m\,c=\frac{1}{n}\,\sum_{i=1}^{n}m\,c_{i}$.

Finally, we define the new latent variable *Z*_*i*_,*i* =  1,…,*n* that indicates the category of interaction groups, i.e., whether the interaction frequency is in the signal or noise group:

$$Z_{i}=\left\{ \begin{array}{c} 1,\mathrm{the}\,\mathrm{pair}\,i\,\mathrm{is}\,a\,\mathrm{signal}\\ 0,\mathrm{the}\,\mathrm{pair}\,i\,\mathrm{is}\,a\,\mathrm{noise} \end{array}\right.$$

The indicator variable has two outcomes (0 and 1), and it follows the Bernoulli distribution, *Z*_*i*_∼Bernoulli(*W*_1*i*_), for *i* =  1, 2,…,*n*, and it is concluded that pair *i* is signal pair whenever *P*(*Z*_*i*_ =  1|*Y*) is bigger than a cut-off value, 0.5 (Niu and Lin, 2015).

### Aggregate Peak Analysis

Aggregate peak analysis is the standard and recommended plot that measures the aggregate enrichment of putative peaks in a contact matrix. It plots the sum of a series of submatrices around the interaction anchors derived from the contact matrix. The matrix is created by summing together all submatrices around each putative individual peak. The resulting APA plot displays the total number of contacts that lie within the entire putative peak set at the center of the matrix. It is recommended to use peak to lower left (P2LL) value to compare the interactions from different methods. We generate an APA plot with 5-kb resolution contact matrices for significant chromatin interactions. The BEDPE files from the ChIA-PET data were used to build interaction matrices.

## Results

Chromatin interaction analysis using mixture model used a mixture model to distinguish signals from noise in the ChIA-PET experiment using the Bayesian approach. To evaluate and compare the performance of ChIAMM with the top existing methods, we used four short and two long-read ChIA-PET datasets. The short reads are RNAPII- and CTCF-associated datasets from human K562 and MCF7 cells, and the long reads are RNAPII- and H3K9me2-associated datasets from rice Minghui 63 (MH63). We used human genome hg19 for K562 and MCF7 datasets and RS1 reference genome for rice datasets.

### Convergence Diagnostics and Posterior Prediction

We used Stan statistical package (rstan) and checked the convergence of the algorithm with the trace plot and Rhat. The rstan package allows us to conveniently fit different models and access the outputs, including posterior inferences. In Bayesian inference, MCMC algorithms will draw a sample from the target posterior distribution after it has converged to equilibrium. However, there is no guarantee about whether it is converged or is close enough to the posterior distribution. Therefore, we have to check its convergence using a trace plot and Rhat. It is well known that trace plots are an essential tool for assessing the mixing of a chain. Trace plot is a time series plot of the Markov chains that shows the evolution of parameter vector over the iterations of one or many Markov chains. The Rhat produces the convergence diagnostic that compares the between- and within-chain estimates for model parameters. It is recommended to run at least four chains by default and use the sample if Rhat is <1.05 (Stan Development Team, 2016). The trace plot of intra- (β_*j*_,λ_0*i*_,*W*_1*i*_, and *C*) and inter- ($\beta_{j},\lambda_{0\,i}^{\prime },W_{1\,i}^{\prime }$, and *C*′) chromosomal interaction model parameters were checked. As we specified in the methodology, the parameters $\lambda_{0\,i},W_{1\,i},\lambda_{0\,i}^{\prime }$, and $W_{1\,i}^{\prime }$ are pair specific. The convergence was checked on the random taken values. Here, as an example, we tested the convergence diagnostic and posterior prediction on MH63 RNAPII datasets. Supplementary Figure 1 and Supplementary Table 1 show the trace plot and Rhat value of the model parameters in the given datasets. The Rhat value of all parameters is 1, and chains are mixed well. Therefore, these results proved to us the convergence of the MCMC algorithm.

Posterior prediction is used to assess the fit between a model and the data. The fitted model has been validated using posterior predictive checks (PPCs) through simulating data from the model using parameters drawn from the posterior. The posterior prediction analysis was checked using a graphical prior and PPC plot. The PPC plot gives the graphical display that compares the observed data to the simulated data from the posterior predictive distribution. In Supplementary Figure 2, the dark line shows the distribution of the observed outcomes, and the lighter line shows the first 100 kernel density estimate from the posterior predictive distribution in the MH63 RNAPII dataset. From the plot, the simulated data is overlapped with the actual data, or we assured that the fitted model recovered the data.

### Comparing the Interactions of Short-Read Data From Different Methods

**FIGURE 2:**
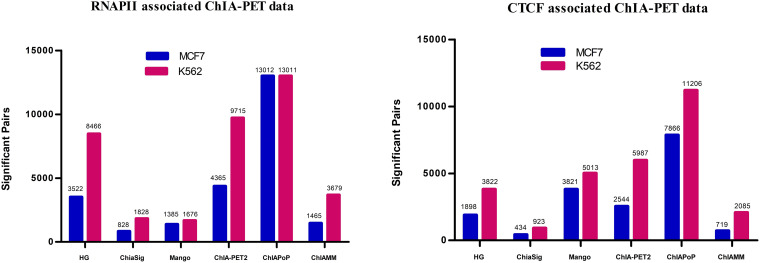
Detected significant interactions in different tools in RNAPII and CTCF data sets. The red and blue vertical bars represent the significant interactions detected in K562 and MCF7 data sets.

In this study, the ChIAMM found significant interactions using the value of *W*_*1i*_ (the probability of pair*i* being a true pair). The significant interactions from HG, ChiaSig, Mango, ChIA-PET2, and ChIAPoP are found using the ChIA-PET Tool (V3), ChiaSig, Mango, ChIA-PET2, and ChIAPoP pipelines, respectively. In all methods, we used the same cut-off of interaction frequency ≥3. ChIAMM detected 1,465 and 3,679 potential pairs in MCF7 and K562 RNAPII datasets, respectively. These significant pairs are more than those identified by ChiaSig (828 in MCF7 and 1,828 in K562) and Mango (1,385 in MCF7 and 1,676 in K562). For CTCF-associated datasets, ChIAMM detected 719 and 2,085 significant pairs in the MCF7 and K562 datasets, respectively, which are more than those identified by ChiaSig (434 in MCF7 and 923 in K562). In contrast, some methods reported more interaction pairs than ChIAMM (Figure 2).

Supplementary Figure 3 shows the overlapped results between ChIAMM and other existing tools. As an example, in the MCF7 RNAPII dataset, we found higher overlapped interactions with HG (1,465), ChiaSig (1,334), and ChIAPoP (1,113). Similarly, in the K562 CTCF dataset, it shows higher overlapped interactions with HG (2,084), ChIAPoP (1,852), and ChiaSig (1,886). Besides, we found 257, 381, 387, and 1,047 overlapped significant interaction pairs among the six tools in MCF7 RNAPII, K562 RNAPII, MCF7 CTCF, and K562 CTCF datasets, respectively.

#### Aggregate Peak Analysis of the Interactions Between Different Methods

We used the APA plots to compare interactions from ChIAMM and other existing methods. To generate APA plots, we built interaction matrices from BEDPE files, and the interaction counts were summed for all pairs of loci in 5-kb bins (Servant et al., 2015). Then, the APA score can quantify the level of a different set of interactions. In the APA plot, it is recommended to use P2LL value for comparison. P2LL is calculated as the ratio of the central pixel to the mean of the pixels in the lower-left corner of the interaction matrices. Higher scores indicate higher enrichment of interaction, and it is always good to find methods with higher P2LL value (Rao et al., 2014). For a fair comparison, in all methods, we considered the significant chromatin interactions with ≥3 supportive PETs. Then, we found the overlapped and unique significant interactions between ChIAMM and other existing tools.

**FIGURE 3:**
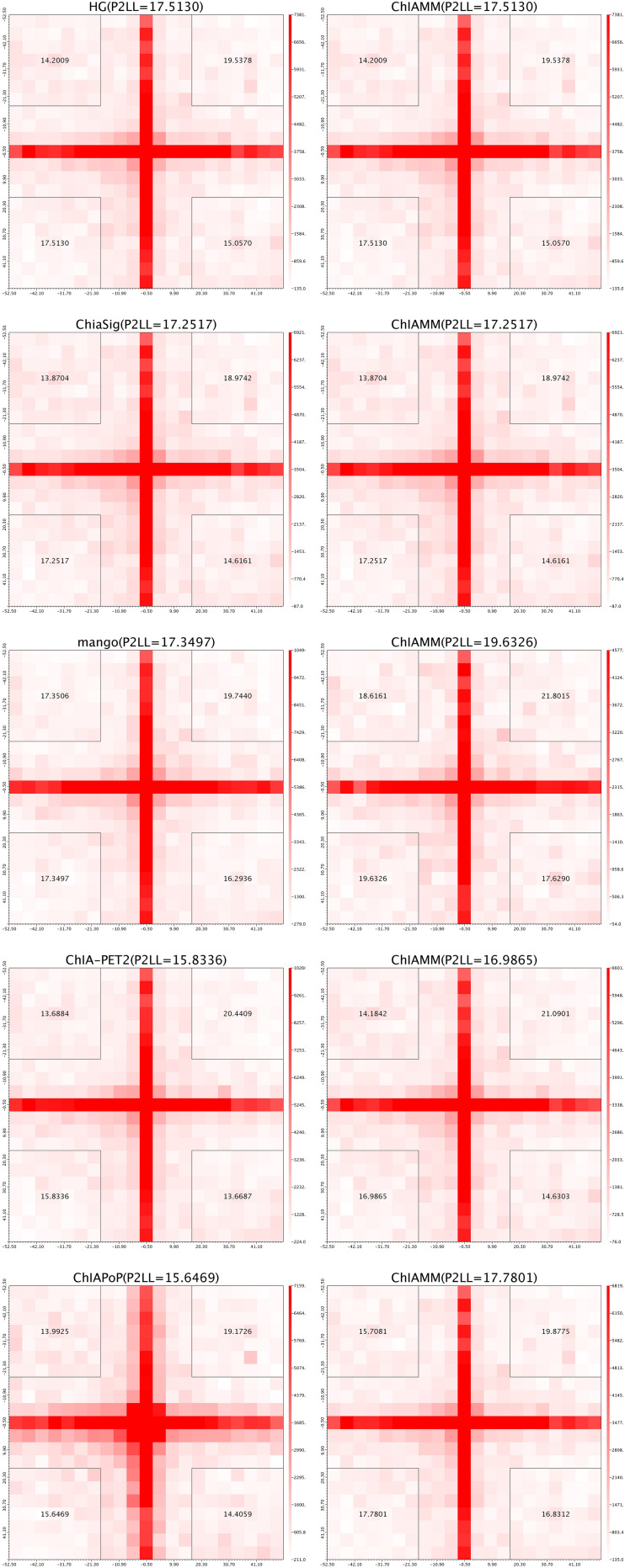
Aggregate peak analysis (APA) plots for overlapped significant interactions between ChIAMM and existing methods in the K562 CTCF ChIA-PET data set. Each row in the plot represents the comparison of interactions between ChIAMM and one other method.

**FIGURE 4:**
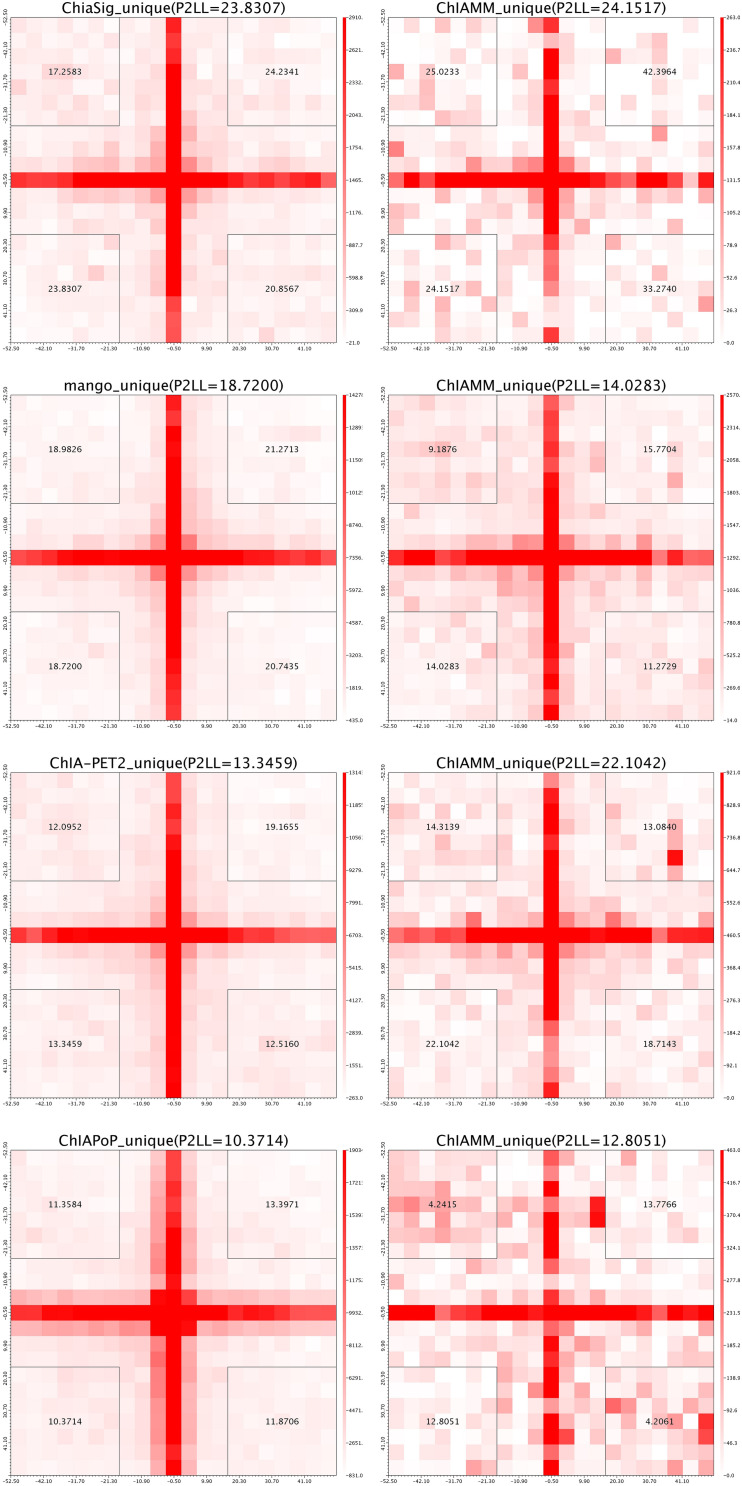
Aggregate peak analysis (APA) plots for significant unique interactions between ChIAMM and existing method in the K562 CTCF ChIA-PET data set. Each row in the plot represents the comparison of interactions between ChIAMM and one other method.

For each dataset, we plotted five pairs of APA plot for overlapped interactions and four pairs of APA plot for unique interactions (no unique interactions found between ChIAMM and HG). In all datasets, in the overlapped interactions, ChIAMM has shown higher P2LL values with other tools. As expected, ChIAMM shows similar P2LL values with HG and ChiaSig tools (Figure 3 and Supplementary Figure 4). Besides, for unique interactions, ChIAMM has shown better pair ranking with other existing methods, with some exceptions, except Mango in K562 RNAPII, Mango in MCF7 and K562 in CTCF, ChiaSig in K562 RNAPII, and ChIAPoP in MCF7 CTCF ChIA-PET datasets (Figure 4 and Supplementary Figure 5).

#### Comparison of CTCF Enrichment for Overlapped and Unique Interactions

In different studies, CTCF is a ubiquitously expressed and essential protein, and the DNA interactions are directly related to this protein (Ohlsson et al., 2010). For comparing enrichment of proteins in anchors, we used different CTCF peak files, i.e., the CTCF-peak regions from ENCODE ChIP-Seq datasets ENCFF720OXG and ENCFF990LUT for MCF7, and ENCFF681OMH and ENCFF559HEE for K562 cell line. For the CTCF coverage computation, we considered the overlapped and unique interactions between ChIAMM and other existing methods with chromatin interaction frequency ≥3. A comparison of CTCF enrichment means how many anchors are covered with the peak file. For both overlapped and unique interactions, we found the anchors that covered with the CTCF peak file. Supplementary Figure 6 shows the percentage of CTCF enriched and non-enriched anchors of the overlapped and unique interactions between ChIAMM and other methods in CTCF associated datasets. In these figures, ChIAMM shows equal CTCF enrichment with HG and ChiaSig in the overlapped interactions and shows a minimal difference with others. To ensure that this difference is statistically significant or not, we computed the Fisher’s exact test. According to the *p*-value, in all datasets, the proportion difference of enriched anchors is statistically insignificant, except for ChIAPoP in the overlapped interactions.

#### Comparison of CTCF Motif Orientation for Overlapped and Unique Interactions

**FIGURE 5:**
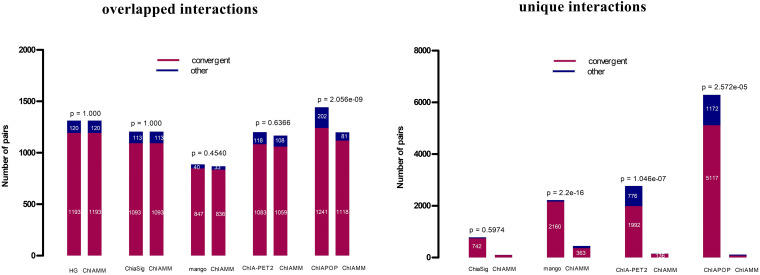
CTCF motif orientation analyses in the K562 CTCF ChIA-PET data set between overlapped and unique interactions in ChIAMM and existing tools. The Fisher’s exact *p*-values are given at each the top of the figure.

It is well known that CTCF is an essential architectural protein to mediate long-range interactions. Different studies have shown that CTCF motif orientations at chromatin loop anchor regions are expected to have more convergent orientation than in other orientations (Zhang et al., 2018). Here, we compared the CTCF motif orientation of significant interactions (intrachromosomal) of ChIAMM with the existing tools. If the interaction is a real signal, it is expected to have convergent orientations more often than in other orientations. For the motif orientation analysis, a webserver https://ccg.epfl.ch/pwmscan/ was used for scanning the reference genome (hg19), and the predicted CTCF motif was filtered and kept only the overlap result with CTCF peak regions. The CTCF peak files are the same as that we used in the previous CTCF enrichment comparison. Then, we found the overlapped result between the filtered predicted CTCF motif and significant chromatin interactions that we found using different tools. After that, we counted the number of significant pairs with convergent and other motif orientations. Figure 5 and Supplementary Figure 7 show the CTCF motif orientation analyses results for the overlapped and unique interactions in K562 and MCF7 CTCF datasets. The red color represents convergent motif orientation, and the blue color represents the other motif orientation. Fisher’s exact *p*-values are given at the top of each bar. The *p*-value shows the test of a proportion of convergent motif orientation between ChIAMM and other existing methods. For each dataset, we performed five and four pairs (no unique interaction between ChIAMM and HG) of CTCF motif orientation analysis for overlapped and unique interactions between ChIAMM and existing methods, respectively. From these plots, in all datasets, ChIAMM showed equal motif orientation with ChiaSig (only in overlapped interactions) and HG. Statistically, the proportional difference in convergent orientation between methods was tested. Based on the *p*-value, in the overlapped interactions, the proportion of ChIAMM motif orientation is not significantly different from other existing approaches, except ChIAPoP. Likewise, in the unique interactions, it is statistically insignificant from others, except for Mango and ChIA-PET2.

### Comparing the Interactions of Long-Read Data From Different Methods

From the existing tools, only ChIA-PET Tool V3 and ChIA-PET2 can analyze long-read ChIA-PET data. Hence, we examined the result of ChIAMM with these two existing tools using the H3K9me2 and RNAPII datasets from rice MH63 variety. We used RS1 as the reference genome. In all methods, for a fair comparison, we considered the interaction frequency ≥3. Similar to the short-read ChIA-PET datasets, we validated the interactions using the APA plot.

Chromatin interaction analysis using mixture model and other existing tools found the different amounts of significant chromatin interactions. Supplementary Figure 8 shows the detected interactions in each tool; besides, it also shows the overlap interactions between ChIAMM and existing tools. HG found maximum significant chromatin interactions (63,745 and 6,242); ChIAMM found the next largest interactions (23,966 and 12,448); and ChIA-PET2 detected the smallest significant chromatin interactions (5,143 and 6,183) in MH63 RNAPII and H3K9me2 datasets, respectively. ChIAMM found maximum overlapped interactions with HG (23,821 and 2,903). The three tools found 2,744 and 969 overlapped significant chromatin interactions in MH63 RNAPII and MH63 H3K9me2 datasets.

#### Aggregate Peak Analysis of the Interactions Between Different Methods

**FIGURE 6:**
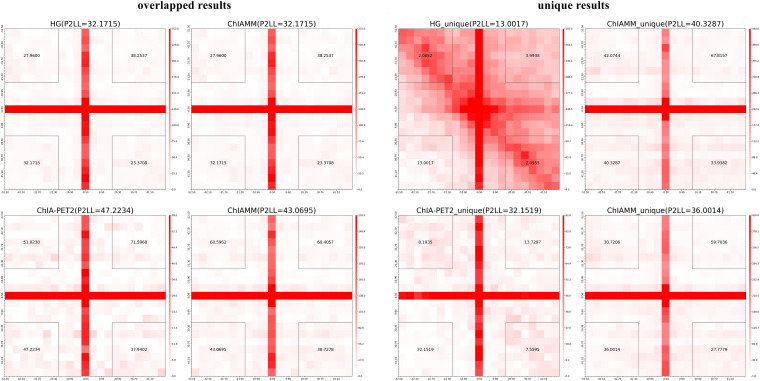
Aggregate peak analysis (APA) plots for overlapped and unique significant interactions between ChIAMM and existing method in rice MH63 H3K9me2 ChIA-PET data set. Each row in the plot represents the comparison of interactions between ChIAMM and one other method.

To compare and evaluate ChIAMM in long-read ChIA-PET datasets, we generated the APA plot. Still, for the sake of fair comparison, we considered the chromatin interaction frequency ≥3. We plotted the APA plots for overlapped and unique significant interactions between ChIAMM and other existing tools. We plotted two pairs of APA plot for overlapped and unique interactions. Figure 6 and Supplementary Figure 9 show the APA plot for overlapped and unique interactions. In unique interactions, ChIAMM has shown higher P2LL values in both datasets. Besides, in the overlapped interactions, ChIAMM shows similar P2LL values with HG and lower P2LL values with ChIA-PET2 in H3K9me2 and RNAPII MH63 datasets.

## Discussion and Conclusion

Chromatin interaction analysis by paired-end tag sequencing is a genome-wide, high-throughput, and high-resolution method to detect chromatin interactions associated with a specific protein of interest. Here, we described a new statistical approach called ChIAMM that corrects for non-specific interactions as a function of genomic distance, enrichment, GC content, and mappability score. It is designed for both short- and long-read ChIA-PET datasets. Using the RNAPII- and CTCF-associated data from human K562 and MCF7 cell and RNAPII- and H3K9me2-associated data from rice Minghui 63 (MH63), we demonstrated that our approach is better with the most effective top existing tools.

In various studies, enrichment, genomic distance, GC content, and mappability score were listed as systematic sources of bias. All the preexisting ChIA-PET tools considered only the genomic distance or enrichment as systematic biases. Therefore, all tools failed to address the possible biases in their study. Some are designed exclusively for short-read and only for intrachromosomal interaction ChIA-PET datasets. In this study, we filled all the above gaps using the Poisson regression model. We considered the genomic distance, enrichment, GC content, and mappability score in the model, and we noticed its effect on the interaction frequency. Supplementary Table 1 shows the estimated Poisson regression coefficients of biases in the MH63 RNAPII dataset. Each bias coefficient has a different sign and magnitude that tells the relationship type (positive or negative) and the degree of its effect, respectively. Enrichment and GC content, and mappability and genomic distance have a positive and negative effect, respectively. Besides, in the intrachromosomal interaction dataset, mappability and enrichment, and in the interchromosomal dataset, the GC content show a higher effect on loop detection.

Furthermore, some tools like Mango examined only intrachromosomal interaction. They removed all interchromosomal interactions in their model because they thought that interchromosomal interactions are the source of biases; besides, they could not find a technique that measures the genomic distance on different chromosomes. In this study, we dealt with these challenges via modeling inter- and intrachromosomal interaction data separately. This technique considered all four biases in the intrachromosomal interaction model and the three biases (we left out the genomic distance) in the interchromosomal interactions model. Using this technique, we salvaged essential significant interchromosomal interactions data rather than removal. Thus, this technique is a novel idea to consider interchromosomal interaction data into the study instead of total eradication.

Supplementary Table S2 shows the significant intra- and interchromosomal interaction (≥3) in various tools. Except for Mango and ChiaSig, other tools detected different amounts of significant interchromosomal interactions. Comparatively, ChIAPoP found the largest interchromosomal interactions; ChIAMM found 24, 28, 24, and 11 significant interchromosomal interactions from MCF7 RNAPII, K562 RNAPII, MCF7 CTCF, and K562 CTCF datasets, respectively. Therefore, discarding all interchromosomal data from the model is not a proper technique. It is considered as removed potential chromatin interaction from the analysis.

We compared ChIAMM results with the other five top existing tools using APA plot, CTCF coverage of anchors, and CTCF motif orientation. In the APA plot, we showed the performance of ChIAMM using overlapped and unique interaction frequency data. In all datasets, ChIAMM showed the highest enrichment of interaction with other existing methods, except Mango, an exceptionally conservative method, and it reports very few chromatin interactions. In the overlapped interactions, ChIAMM showed equal P2LL values with HG and ChiaSig, as expected, because ChIAMM and ChiaSig used ChIA-PET Tool as a primary processing pipeline, and this is also true for CTCF coverage and CTCF motif orientation analysis results. In CTCF coverage and motif orientation analysis, the new approach showed equal CTCF coverage and motif orientation with HG and ChiaSig in the overlapped interactions and relatively minimal differences with others. However, in almost all comparisons, the difference is statistically insignificant.

We compared the running time of ChIAMM with other preexisting methods. As an example, we analyzed the MCF7 CTCF ChIA-PET with threads, 12; RAM, 64 GB; cluster operating system, CentOS 6.6; central processing unit, Intel(R) Xeon(R) CPU E5-2680 v3 @ 2.50 GHz. ChIAMM took 48.1 min and showed better performance. ChIA-PET Tool, ChiaSig, Mango, ChIA-PET2, and ChIAPoP took 17, 37, 36, 31, and 23 h, respectively. Overall, ChIAMM is the outperformed novel, fastest, and user-friendly tool than the most existing methods.

## Data Availability Statement

The datasets presented in this study can be found in online repositories. The names of the repository/repositories and accession number(s) can be found in the article/ Supplementary Material.

## Author Contributions

YA and GL: conceptualization. YA: methodology and writing original draft preparation. YA and HJ: software. YA, SW, and JZ: data analysis. YA, GL, SW, and JZ: review and editing. GL and XN: supervision. GL: funding acquisition. All authors contributed to the article and approved the submitted version.

## Source Code and Documentation

The source code and documentation for the ChIAMM is available at http://www.guolianglab.org/subpages/RESOURCES/softwares.php or https://github.com/Yab29/ChIAMM.

## Conflict of Interest

The authors declare that the research was conducted in the absence of any commercial or financial relationships that could be construed as a potential conflict of interest.
