# Supplementary material for: ChIAMM: A Mixture Model for Statistical Analysis of Long-Range Chromatin Interactions From ChIA-PET Experiments
Source: Front Genet. 2020 Dec 14;11:616160. doi: 10.3389/fgene.2020.616160 (PMC7767989; doi:10.3389/fgene.2020.616160)
Supplement: Supplementary file 1 [file Data_Sheet_1.pdf]

Supplementary Material

1 Data preparation

The variables in the study are the chromatin interaction loops, the genomic distance between anchors, tag counts in the anchor regions, self-ligation PETs, mappability, and GC content. The chromatin interaction loops, the genomic distance between anchors, tag counts in the anchor regions, and self-ligation PETs are obtained using ChIA-PET Tool (V3) without any FDR cutoff value. The average tag counts in the anchor regions and self-ligation PETs of each anchor are used to measure the marginal count and the enrichment of anchors, respectively. For short-read ChIA-PET, the 35mer window size uniqueness mappability score is downloaded from the ENCODE page http://genome-asia.ucsc.edu/cgi-bin/hgFileUi?db=hg19&g=wgEncodeMapability. For long-read ChIA-PET, we prepared a 35mer window size uniqueness mappability score using ngs-tools. Finally, the GC content of anchors is calculated using the bedtools *nuc* command.

2 Supplementary Figures and Tables

2.1 Supplementary Figures

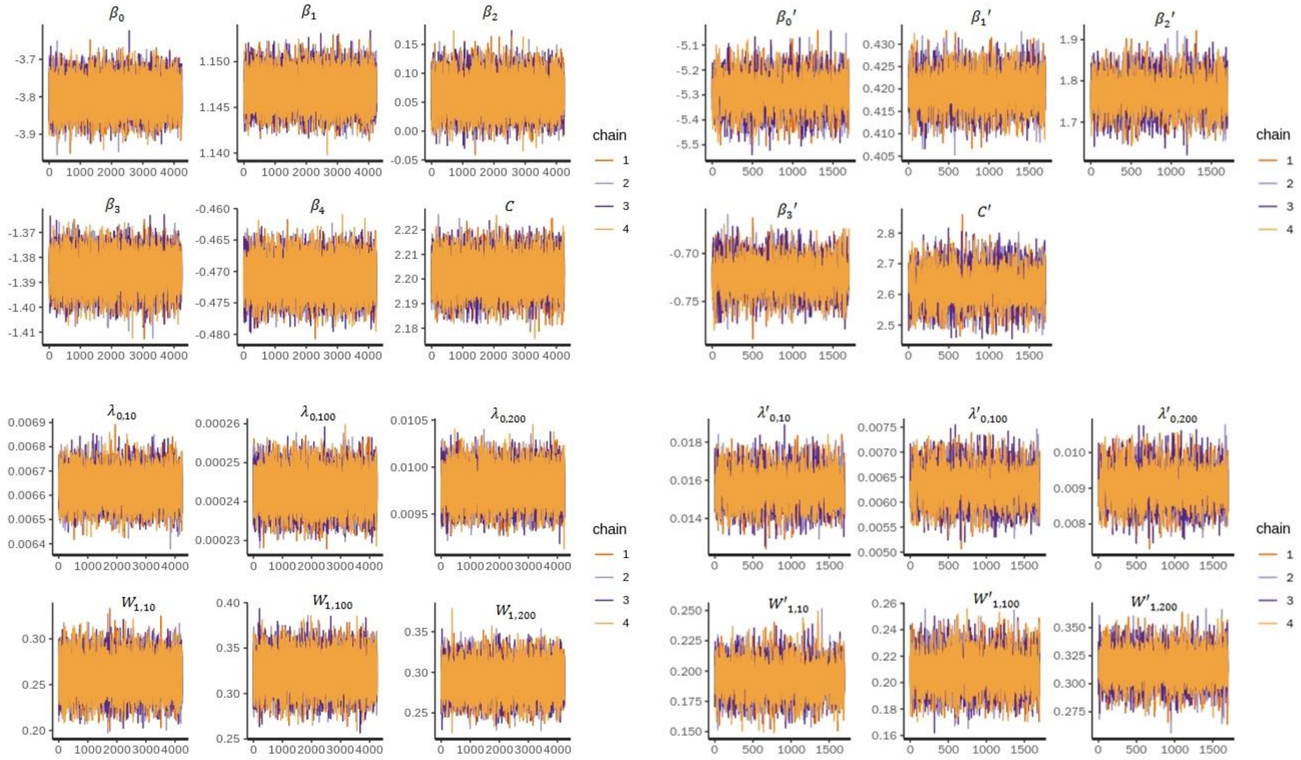

Supplementary Figure 1. Trace plots for MH63 RNAPII data sets**.** Trace plots of MH63 RNAPII ChIA-PET data sets. The parameters $\lambda_{0i}, W_{1i}, \lambda'_{0i}, W'_{1i}$ are pair specific. Hence, we checked the convergence on random taken values. The trace plots show the well-mixed chains that approved the convergence of the MCMC algorithm.

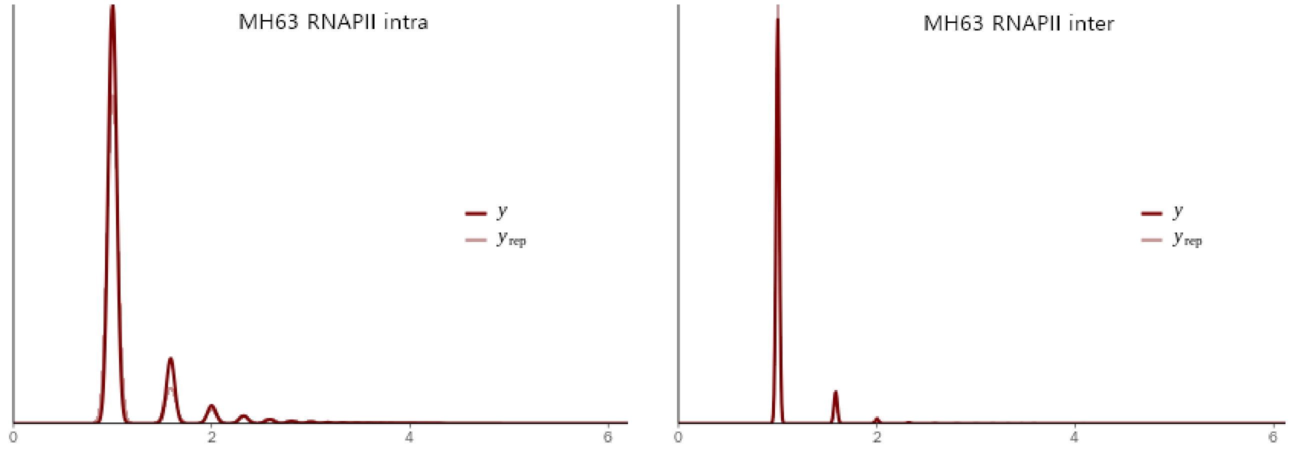

Supplementary Figure 2. Posterior predictive check (PPC) plots for MH63 RNAPII data sets. The PPC plots were created using bayesplot, and the actual data ($log_2(y)$) were compared with the simulated data ($log_2(y_{rep})$). The real value is overlapped with the simulated data, which assures that the model recovered the data very well.

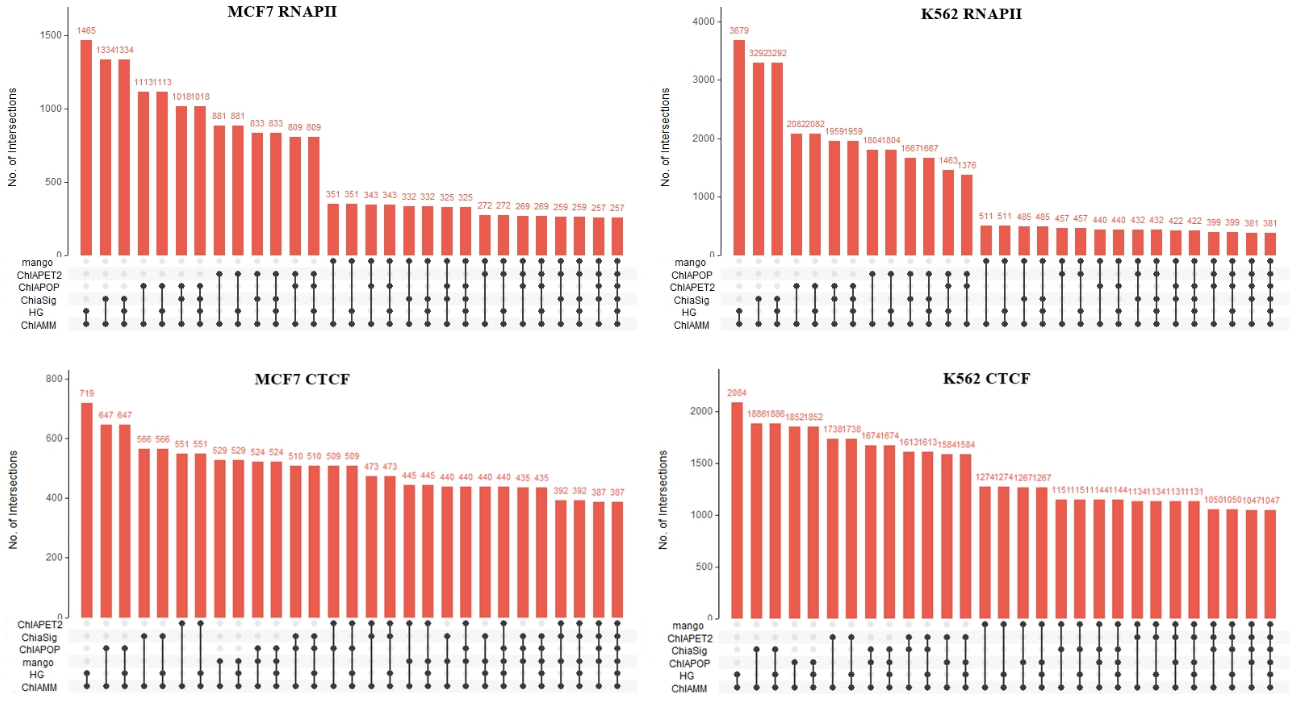

Supplementary Figure 3. Overlapped significant interactions between different tools. UpSet plots of RNAPII and CTCF associated ChIA-PET data sets. The vertical bars represent the sizes of various intersections of significant interactions. The intersections that correspond to a vertical bar are specified by the vertical black line with black filled circles under the bar.

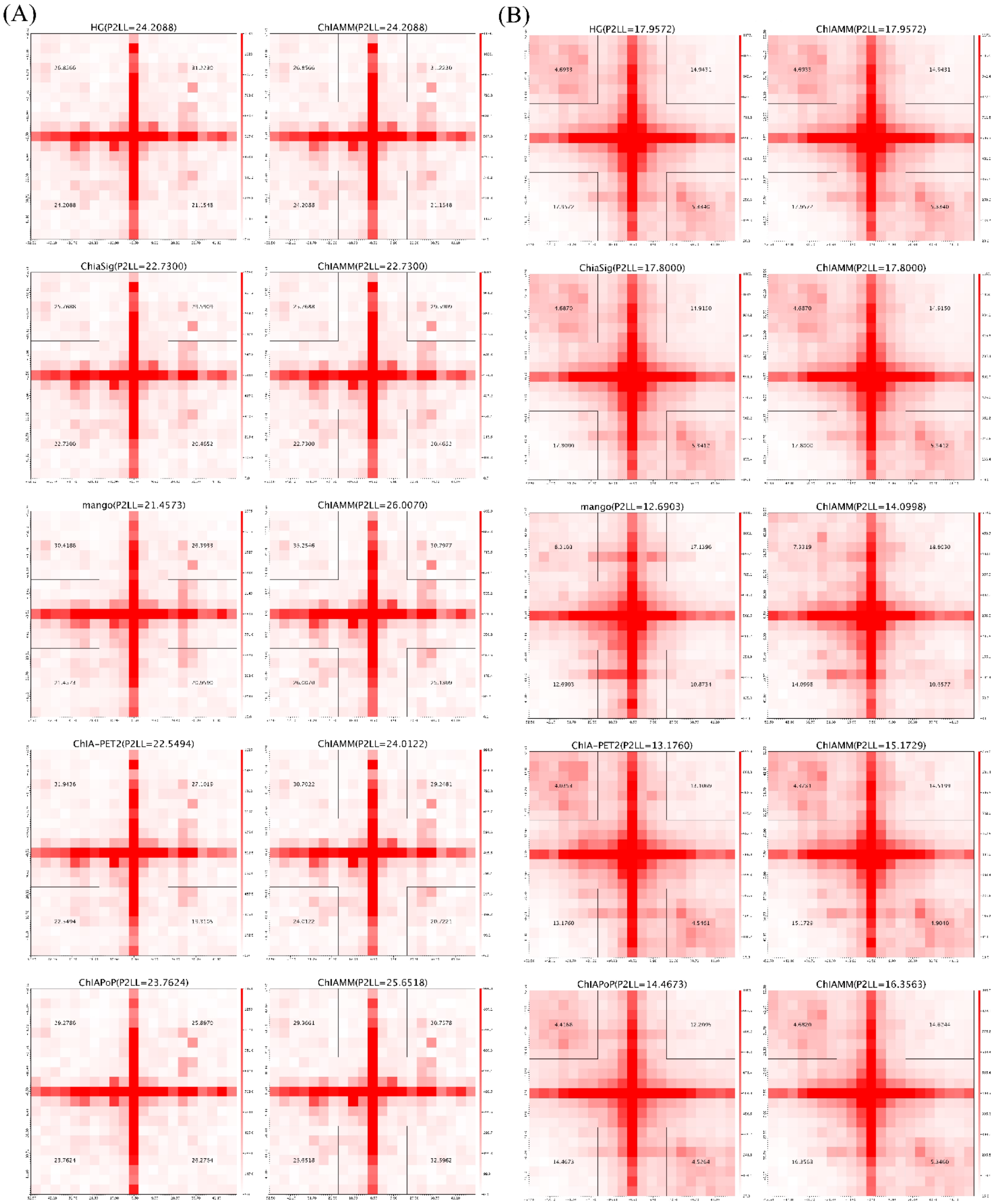

(A)
HG(P2LL=24.2088)
ChIAMM(P2LL=24.2088)
ChiaSig(P2LL=22.7300)
ChIAMM(P2LL=22.7300)
mango(P2LL=21.4573)
ChIAMM(P2LL=26.0070)
ChIA-PET2(P2LL=22.5494)
ChIAMM(P2LL=24.0122)
ChIAPoP(P2LL=23.7624)
ChIAMM(P2LL=25.6518)
(B)
HG(P2LL=17.9572)
ChIAMM(P2LL=17.9572)
ChiaSig(P2LL=17.8000)
ChIAMM(P2LL=17.8000)
mango(P2LL=12.6903)
ChIAMM(P2LL=14.0998)
ChIA-PET2(P2LL=13.1760)
ChIAMM(P2LL=15.1729)
ChIAPoP(P2LL=14.4673)
ChIAMM(P2LL=16.3563)

(C)

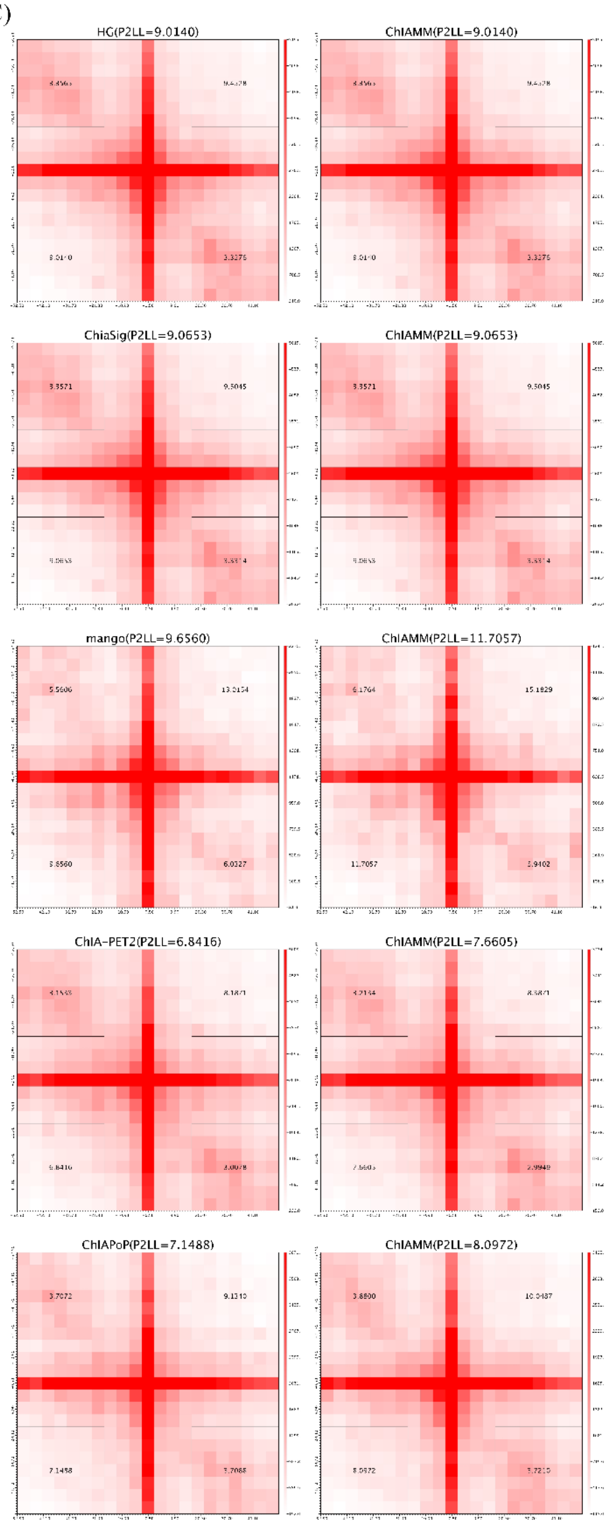

Supplementary Figure 4. APA plots for overlapped interactions in CTCF and RNAPII data sets. APA for overlapped significant interactions between ChIAMM and existing methods in MCF7 CTCF (**A**), MCF7 RNAPII (**B**), and K562 RNAPII (**C**) ChIA-PET data sets. From each plot, the P2LL value is given at the top, and a higher P2LL indicates a better validation. Each row in the sub-plot represents the comparison of interactions between ChIAMM and one other method.

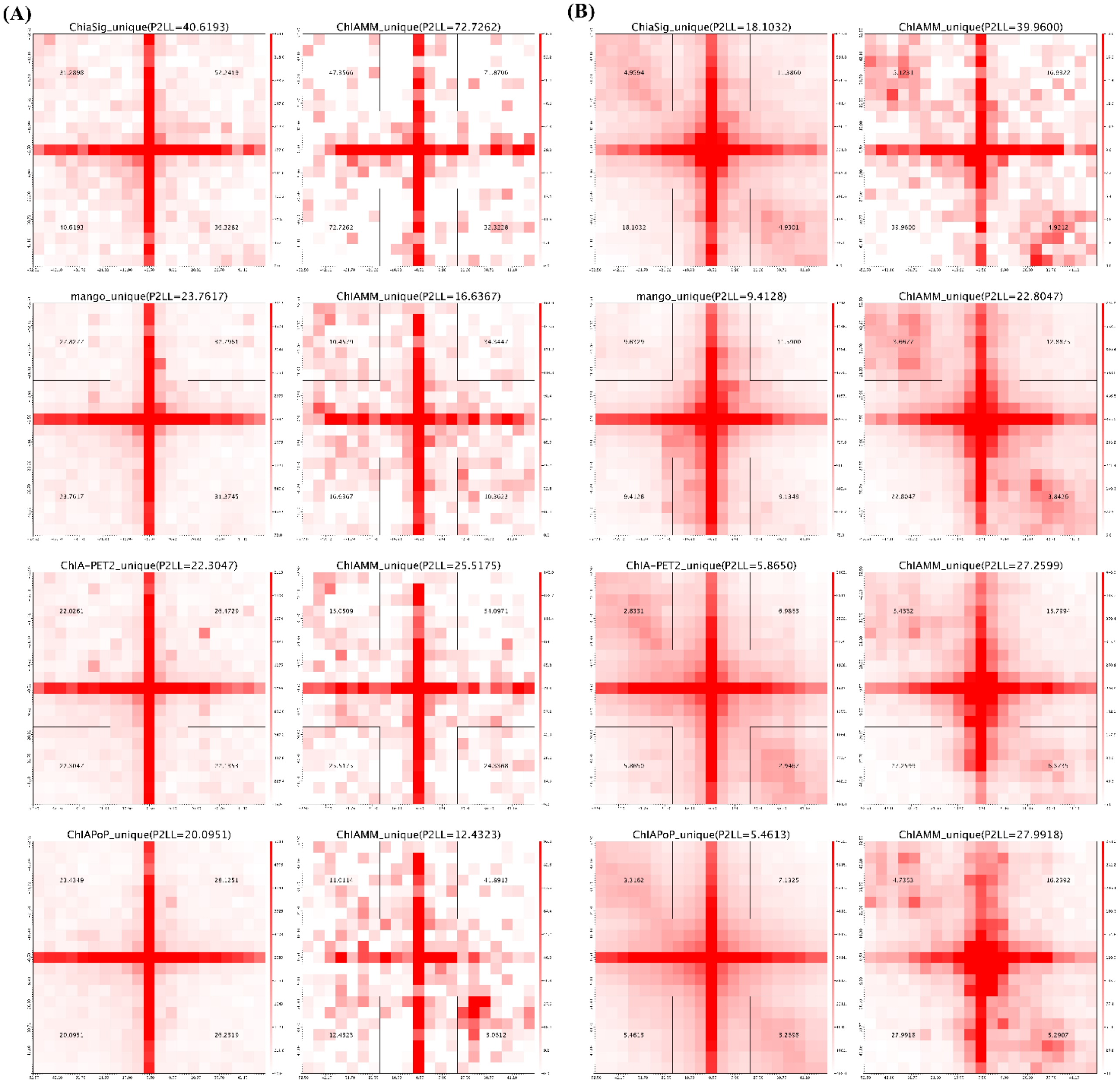
(A)
ChiaSig_unique(P2LL=40.6193)
ChIAMM_unique(P2LL=72.7262)
mango_unique(P2LL=23.7617)
ChIAMM_unique(P2LL=16.6367)
ChIA-PET2_unique(P2LL=22.3047)
ChIAMM_unique(P2LL=25.5175)
ChIAPoP_unique(P2LL=20.0951)
ChIAMM_unique(P2LL=12.4323)
(B)
ChiaSig_unique(P2LL=18.1032)
ChIAMM_unique(P2LL=39.9600)
mango_unique(P2LL=9.4128)
ChIAMM_unique(P2LL=22.8047)
ChIA-PET2_unique(P2LL=5.8650)
ChIAMM_unique(P2LL=27.2599)
ChIAPoP_unique(P2LL=5.4613)
ChIAMM_unique(P2LL=27.9918)

(C)

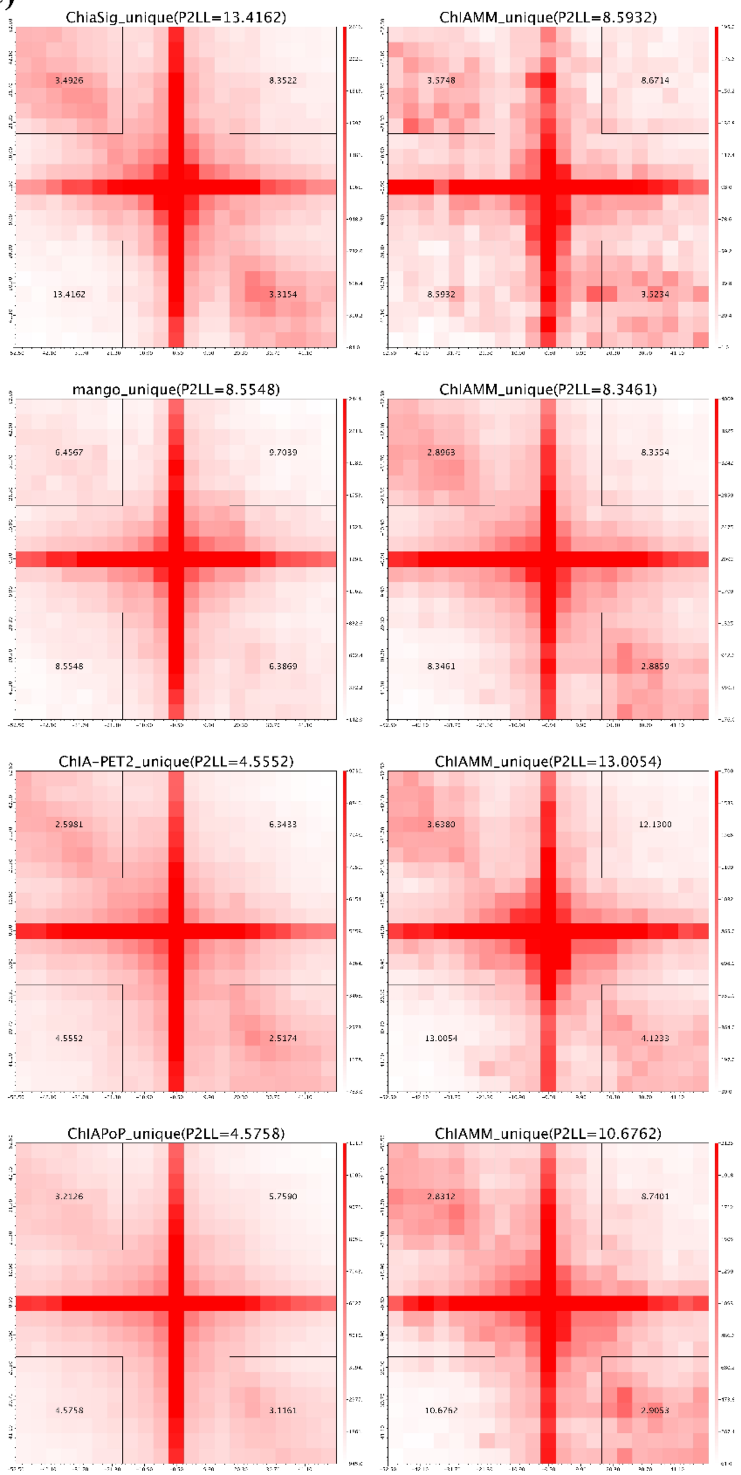

Supplementary Figure 5. APA plots for unique interactions in CTCF and RNAPII data sets. APA for significant unique interactions between ChIAMM and existing methods in MCF7 CTCF (**A**), MCF7 RNAPII (**B**), and K562 RNAPII (**C**) ChIA-PET data sets. From each plot, the P2LL value is given at the top, and a higher P2LL indicates a better validation. Each row in the sub-plot represents the comparison of interactions between ChIAMM and one other method.

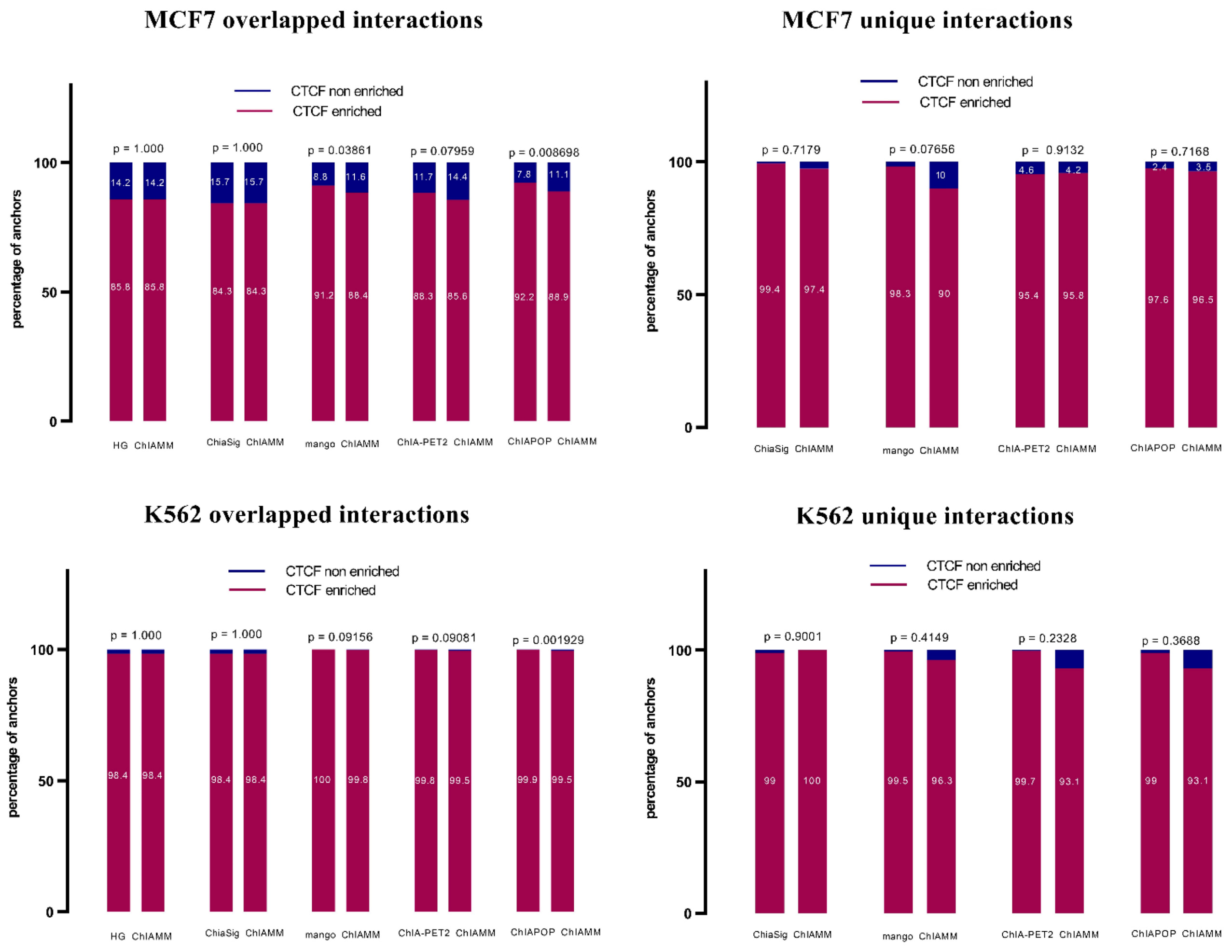

Supplementary Figure 6. CTCF coverage of overlapped and unique interactions in the CTCF ChIA-PET data sets. CTCF enrichment of anchors in MCF7 and K562 CTCF ChIA-PET data set between overlapped and unique interactions in ChIAMM and existing methods. The Fisher exact P-values shown in the figures are for the test of proportions of CTCF enriched between ChIAMM and existing methods.

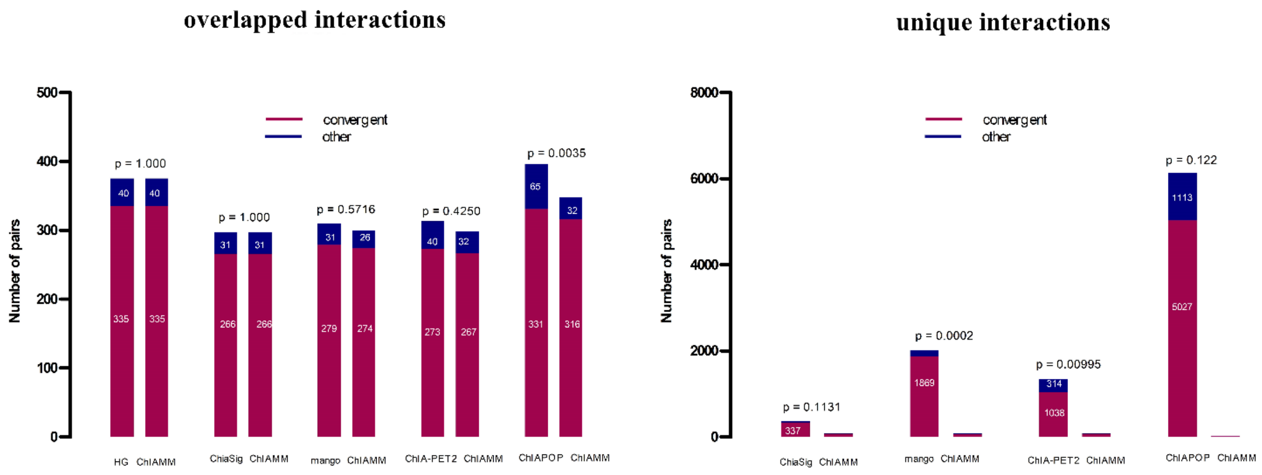

Supplementary Figure 7. Motif orientation analyses in the MCF7 CTCF data set. CTCF motif orientation analyses of overlapped and unique interactions between ChIAMM and existing methods in the MCF7 CTCF data set. The Fisher exact P-values are given in each top of the figure that shows the tests of proportions of motifs with convergent orientation between the ChIAMM and existing methods.

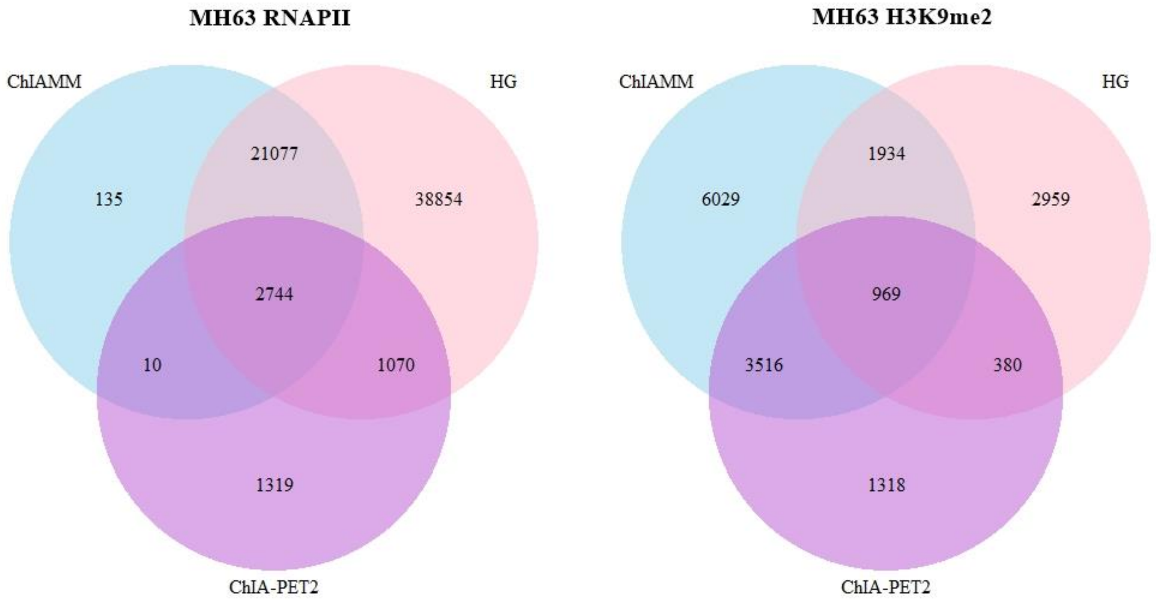

Supplementary Figure 8. Venn diagram of MH63 RNAPII and H3K9me2 data sets. Venn diagram shows the significant interactions between ChIAMM and existing tools in MH63 RNAPII and H3K9me2 ChIA-PET data sets.

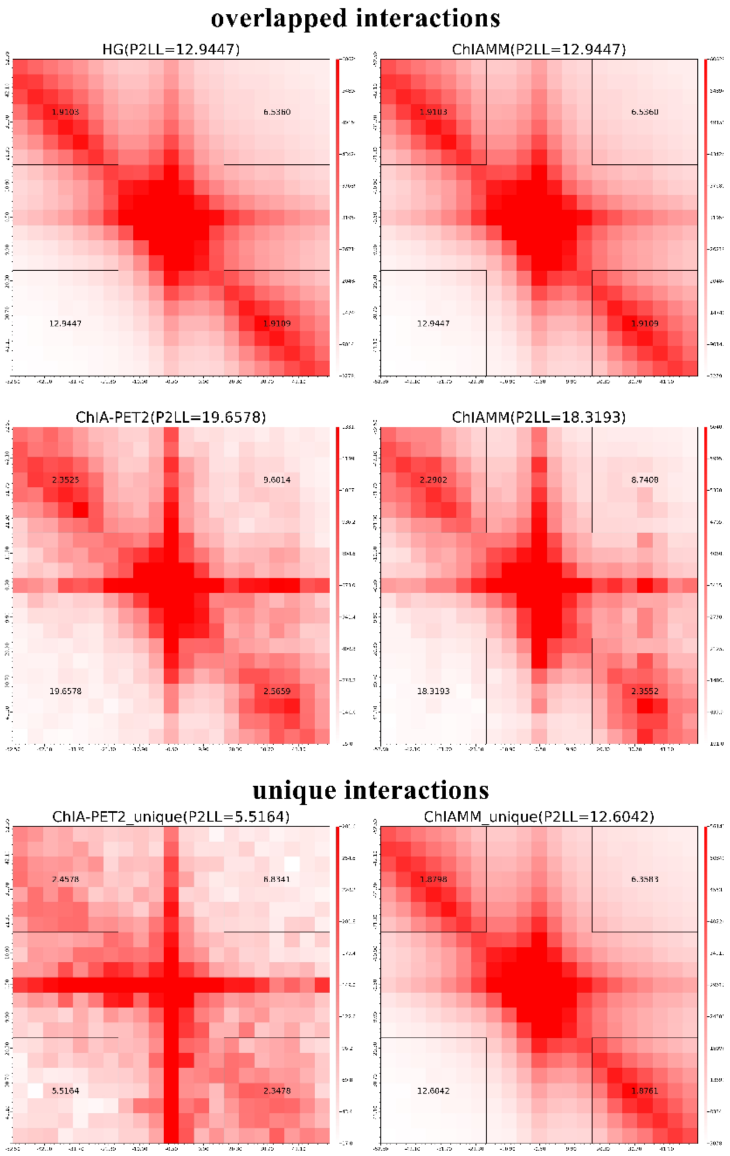

Supplementary Figure 9. APA plot for significant interaction in the MH63 RNAPII data set. APA plot for overlapped and unique significant interactions between ChIAMM and existing method in the MH63 RNAPII ChIA-PET data set. From each plot, the P2LL value is given at the top, and a higher P2LL indicates a better validation. Each row in the sub-plot represents the comparison of interactions between ChIAMM and one other method.

2.2 Supplementary tables

Supplementary Table 1. Summary result of MH63 RNAPII ChIA-PET data sets. The summary result shows the Poisson regression coefficients and the sampler diagnostics in MH63 RNAPII data sets. We run 2,000 and 5,000 iterations for inter and intra data set with a 15% warmup for all data sets. $Rhat$ shows the convergence diagnostic that compares the between- and within-chain estimates for model parameters. If the value of $Rhat$ is smaller than 1.05, it implies that the chains are mixed and converged well. In all data sets, the parameter $Rhat = 1$, which approved the convergence of the MCMC algorithm.

Intra-chromosomal				Inter-chromosomal			
Coef.	mean	n_eff	Rhat	Coef.	mean	n_eff	Rhat
β_0	-3.799	17151	1	$\beta_0{}'$	-5.292	4167	1
β_1	1.147	21022	1	$\beta_1{}'$	0.420	7759	1
β_2	0.063	20798	1	$\beta_2{}'$	1.769	6264	1
β_3	-1.386	32786	1	$\beta_3{}'$	-0.724	9700	1
β_4	-0.471	26677	1	C'	2.628	5073	1.001
C	2.202	27161	1				
Note: β_0 and $\beta_0{}'$ represent the constants, β_1 and $\beta_1{}'$ represent the coefficient of enrichment, β_2 and $\beta_2{}'$ represent the coefficient of GC content, β_3 and $\beta_3{}'$ represent the coefficient of mappability and β_4 represents the coefficient of genomic distance							

Supplementary Table 2. Significant intra- and inter-chromosomal interactions. Significant intra- and inter-chromosomal interactions (≥ 3) were detected in different tools in RNAPII and CTCF ChIA-PET data sets.

Tools	MCF7 RNAPII		K562 RNAPII		MCF7 CTCF		K562 CTCF	
	Intra	Inter	Intra	Inter	Intra	Inter	Intra	Inter
HG	3478	44	8424	42	1866	32	3807	15
ChiaSig	828	-	1825	-	434	-	923	-
Mango	1385	-	1676	-	3821	-	5013	-
ChIA-PET2	4288	77	9657	58	2528	16	5979	8
ChIAPoP	12237	775	12502	509	7689	177	11009	197
ChIAMM	1441	24	3651	28	695	24	2074	11
